# COVID-19 Rebound After Paxlovid Treatment: A Case Series and Review of Literature

**DOI:** 10.7759/cureus.26239

**Published:** 2022-06-23

**Authors:** Shatha Alshanqeeti, Ashish Bhargava

**Affiliations:** 1 Internal Medicine, Ascension St. John Hospital, Detroit, USA; 2 Infectious Disease, King Saud University, Riyadh, SAU

**Keywords:** covid-19, protease inhibitor, paxlovid, nirmatrelvir, ritonavir

## Abstract

Since the declaration of COVID-19 as a pandemic in 2020, several therapies have been developed to reduce symptoms of COVID-19 infection and prevent progression. Paxlovid is an antiviral that was authorized for emergency use in December 2021 for non-hospitalized symptomatic patients with COVID-19 to prevent progression to severe disease. Relapse of symptoms following a period of improvement after treatment with Paxlovid has been described recently. Data are limited, but the disease course in available case reports is usually mild and requires no additional antiviral treatment. We present the cases of COVID-19 relapse (COVID-19 rebound) in two patients following treatment with Paxlovid.

## Introduction

SARS-CoV-2 is a coronavirus that was first described in 2019 and is the cause of the coronavirus disease 2019 (COVID-19) pandemic. Since its declaration as a pandemic in March 2020, tremendous efforts have been put into developing therapies. Multiple vaccines have shown high efficacy; however, breakthrough infections have been reported [1]. Developing therapies have proven to be important in order to prevent severe infection especially in individuals who have a high risk of progression to severe disease. Current therapies include neutralizing antibodies targeting M spike protein and antivirals targeting vital steps in viral replication [2]. On December 22, 2021, an emergency use authorization (EUA) was issued by the United States Food and Drug Administration (FDA) for the oral main protease (M pro) inhibitor nirmatrelvir and ritonavir to be used in the treatment of patients with mild-to-moderate COVID-19 who are at a risk of progression to severe disease [3]. After some cases of recurrence of COVID-19 symptoms (COVID-19 rebound) two to eight days following treatment with Paxlovid, a healthcare advisory was issued by the Centers for Disease Control and Prevention (CDC) on May 24, 2022 [4]. We present two cases of COVID-19 rebound following treatment with ritonavir-boosted nirmatrelvir.

## Case presentation

Case 1

**Figure 1:**
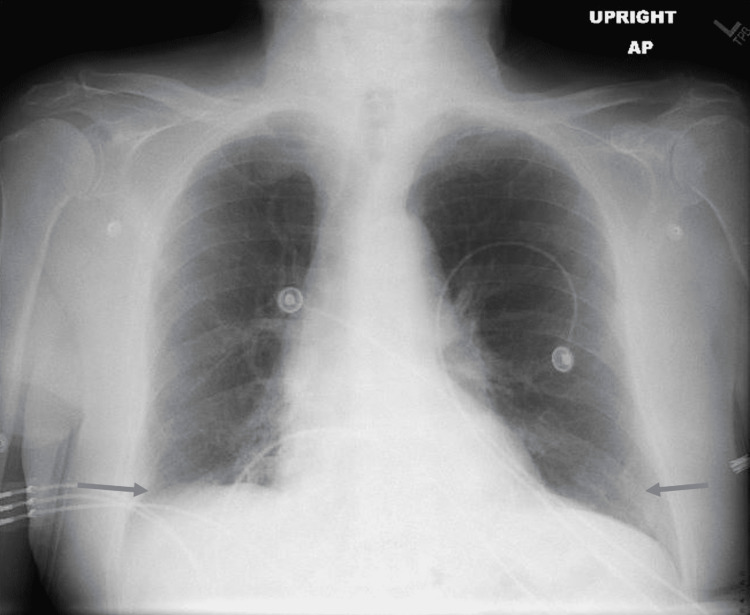
Chest X-ray showing bibasilar opacities

**Figure 2:**
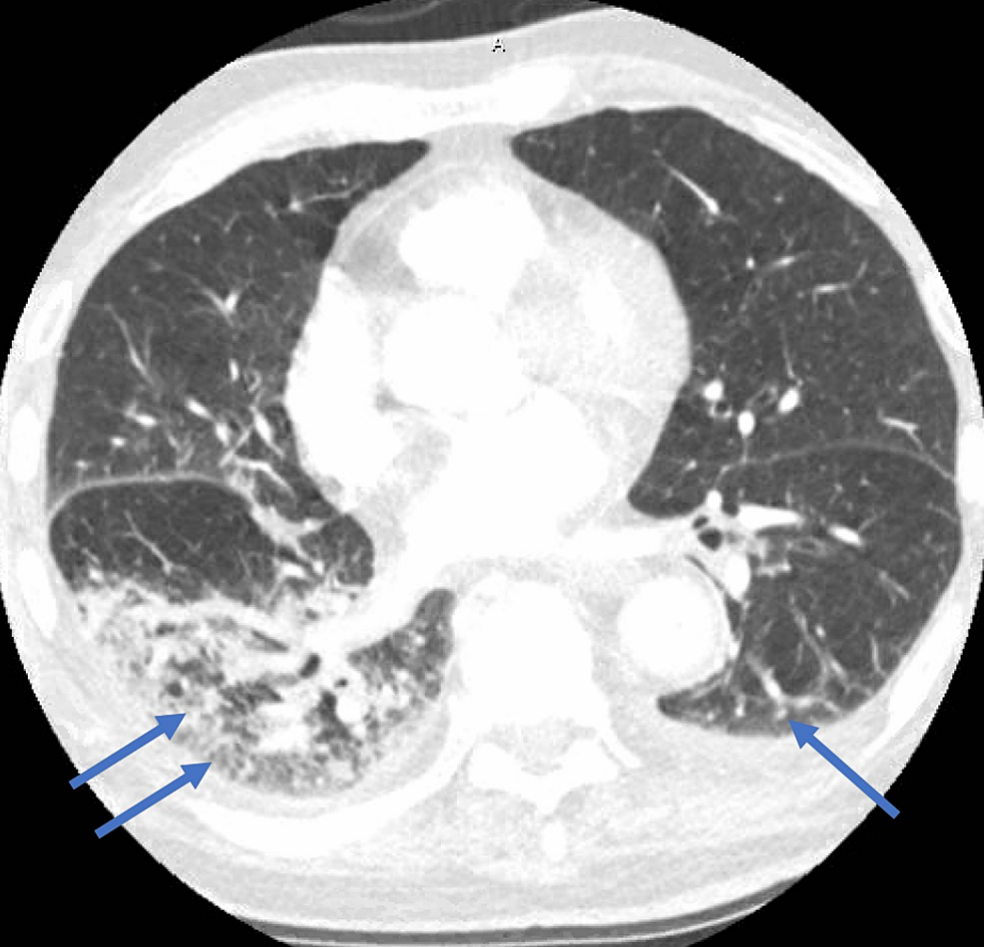
CT angiography of the chest showing acute pulmonary infiltrates in lower lobes

An 87-year-old male with a past medical history of hypertension and rheumatoid arthritis, not on immune suppression therapy, who has been vaccinated against COVID-19 was diagnosed with COVID-19 at his nursing home after having symptoms of shortness of breath, cough, fatigue, and myalgia. He received two doses of Pfizer-BioNTech COVID-19 vaccine; the second dose was administered around 1 year prior to his COVID-19 diagnosis. On day 0 of COVID-19 diagnosis, he received Paxlovid 300-100 mg twice daily for five days with improvement of his symptoms following the completion of the treatment course. He continued to have fatigue and started to develop worsening shortness of breath on day 11 of his diagnosis. He was requiring 2 L of oxygen at his nursing home and was sent to the emergency department. On presentation, the patient’s temperature was 37 degrees Celsius, blood pressure was 117/59 mmHg, heart rate 89 was bpm, oxygen saturation was 96% on 3 L of oxygen, and respiratory rate was 18 breaths per minute. His physical examination was within normal limits, and his body mass index was 22.8 kg/m^2^. His labs were significant for leukocytosis of 18.52 K/mcL with an absolute lymphocyte count of 0.74 K/mcL, thrombocytosis of 434 K/mcL, elevated D-dimer of 560 ngFEU/mL, creatinine of 0.8 mg/dL, C-reactive protein (CRP) of 102.8 mg/L, lactic acid of 1.2 mmol/L, and procalcitonin of 0.09 ng/mL. Nasal SARS-CoV-2 reverse-transcriptase polymerase chain reaction (RT-PCR) was positive. Two sets of blood cultures were drawn and were negative. Chest X-ray (Figure 1) was performed, which showed bibasilar opacities. CT angiography of the chest (Figure 2) showed acute bilateral pulmonary infiltrates in the lower lobes with no evidence of a pulmonary embolism. He was started on symptomatic management with antipyretics, antitussive medications, bronchodilators, and zinc. Oxygen supplementation was weaned off, and his oxygen saturation was 98% on room air. White blood cell (WBC) count improved to 12.9 K/mcL and CRP went down to 47.5 mg/L. The patient’s symptoms improved by day 19 of his diagnosis, and he was discharged to subacute rehabilitation.

Case 2

**Figure 3:**
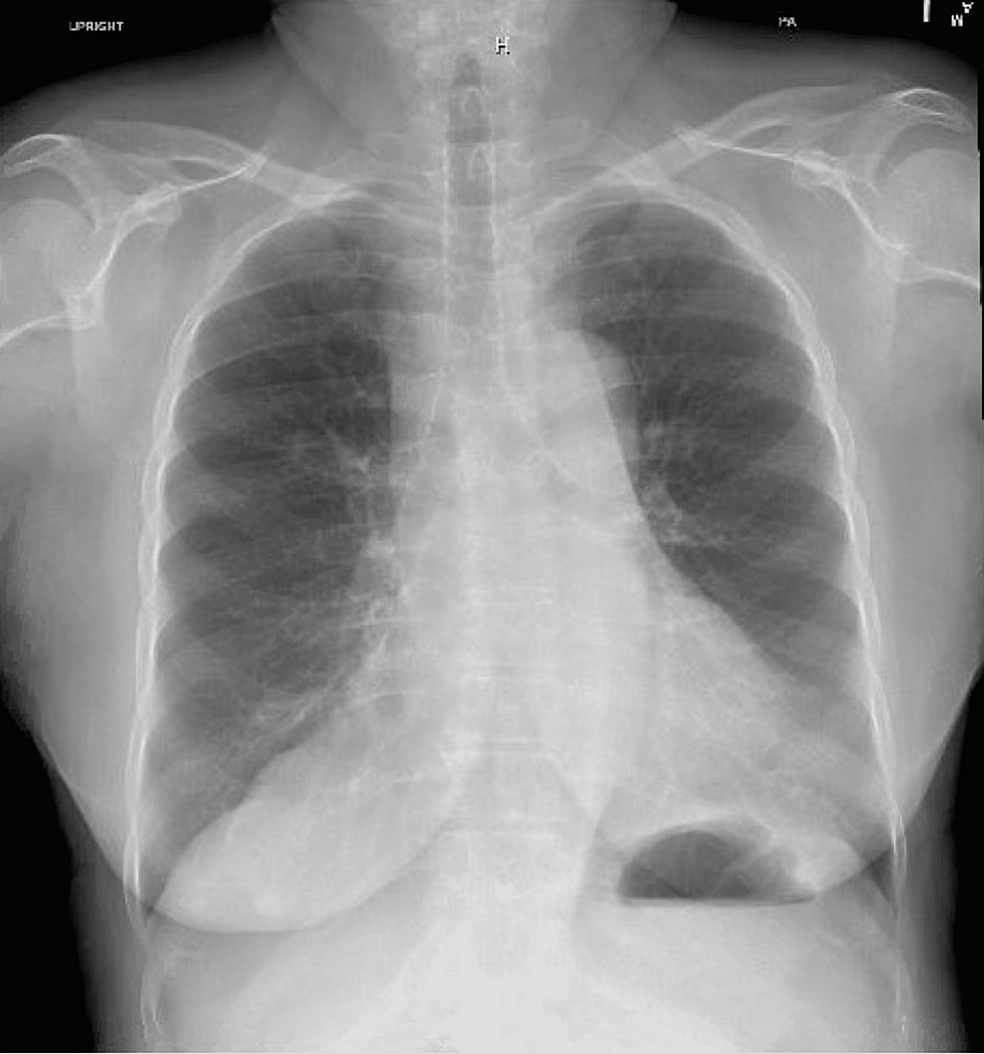
Normal chest X-ray

**Figure 4:**
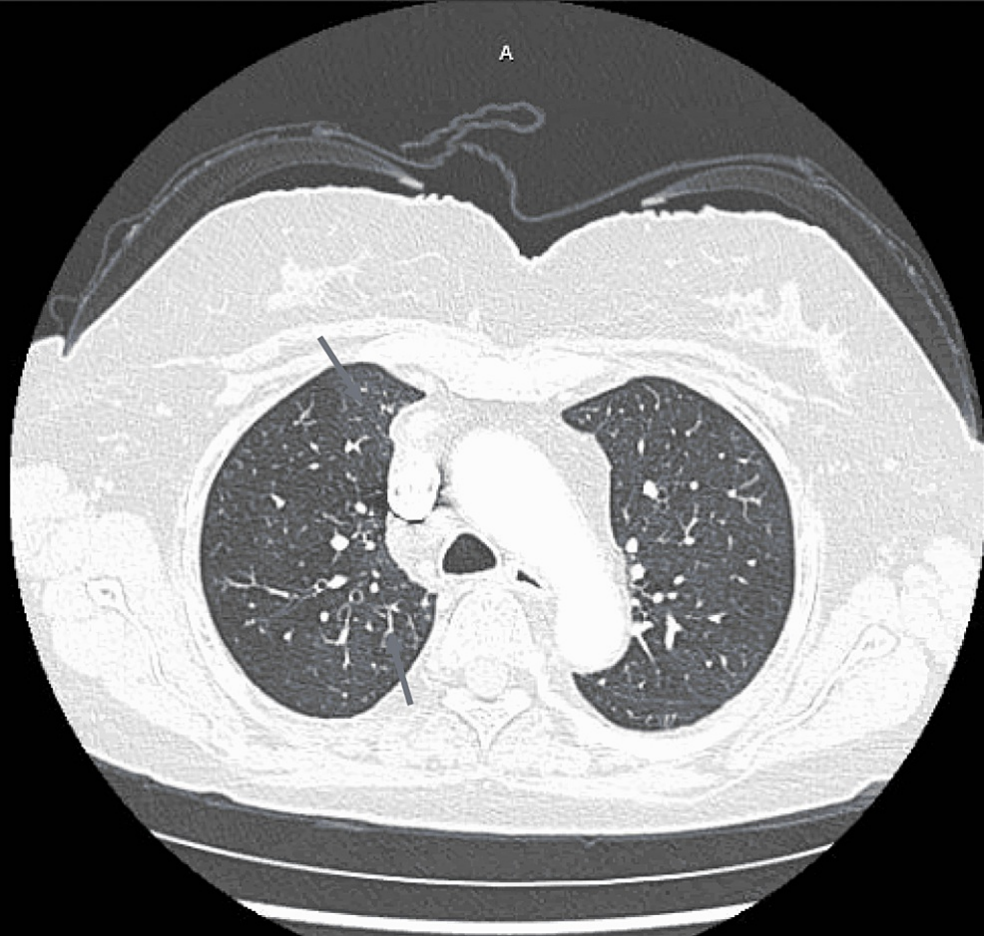
CT angiography showing diffuse patchy opacities in the right lung

A 72-year-old female with a past medical history of rheumatoid arthritis, not on immune suppression therapy, anxiety, and transient ischemic attack, and vaccinated against COVID-19 was diagnosed with COVID-19 after having symptoms of shortness of breath, cough, and fatigue. She received two doses of COVID-19 Moderna vaccine; her second dose was 14 months prior to her COVID-19 diagnosis. The patient was started on Paxlovid 300-100 mg twice daily for five days on day 0 of her diagnosis. Her symptoms improved while on Paxlovid but then started to progressively get worse. She had worsening shortness of breath, fatigue, and started developing pleuritic chest pain and palpitations. On day 14 of her COVID-19 diagnosis, she had episodes of hypoxia at home with oxygen saturation down to 80%; she called her primary care physician who ordered lab work and imaging. Her labs showed a WBC count of 21.92 K/mcL with an absolute lymphocyte count of 1.32 K/mcL, platelet count of 358 K/mcL, creatinine level of 0.82 mg/dL, CRP level of 18.4 mg/L, and procalcitonin level was 0.08 ng/mL. Chest X-ray was within normal limits (Figure 3). She was sent to the emergency department on day 17 of her COVID-19 diagnosis after her D-dimer was found to be elevated at 1,750 ngFEU/mL and her CT showed diffuse patchy opacities in the right lung with no evidence of pulmonary embolism (Figure 4). On presentation to the emergency department, her temperature was 37 degrees Celsius, blood pressure was 129/88 mmHg, she was tachycardic with a heart rate of 127 bpm, respiratory rate was 16 breaths per minute, and oxygen saturation was 100% on room air. On physical examination, she was tachycardic and without distress. Her breath sounds were equal, abdominal and lower extremity examinations were within normal limits, and body mass index was 22 kg/m^2^. She had a respiratory viral panel done, which came back negative. SARS-CoV-2 RT-PCR was performed, which was positive. She was started on symptomatic and supportive management with antipyretics, antitussive medications, bronchodilators, and zinc. Her symptoms improved, and she was discharged home on day 20 of COVID-19 diagnosis.

## Discussion

SARS-CoV-2 is a coronavirus that infects the respiratory tract of humans and animals. It was first described in 2019 and has been implicated in the development of the COVID-19 pandemic declared in March of 2020. Therapies targeted at enzymes involved in viral replication have been developed since then. Paxlovid is an oral antiviral that contains nirmatrelvir and ritonavir. Nirmatrelvir is a main protease (Mpro) inhibitor that has activity against all coronaviruses that infect humans; it acts by interfering with viral replication. Ritonavir is a potent cytochrome P450 (CYP) 3A4 that is co-packaged with nirmatrelvir to inhibit its metabolism and boost its activity [5]. A double-blind randomized controlled trial (EPIC-HR) compared the risk of progression in symptomatic unvaccinated COVID-19 patients who received nirmatrelvir with ritonavir to placebo. Results showed an 88.9% relative risk reduction in hospitalization for COVID-19 or death at day 28 in patients receiving nirmatrelvir with ritonavir compared to placebo [6]. In December of 2021 Paxlovid was made available under EUA after the EPIC-HR trial results were available. It is only used to prevent hospitalization and progression to severe disease in individuals at a high risk of progression [3]. Medical conditions that were found to be associated with a high risk of progression to severe disease included malignancy, diabetes mellitus, chronic lung disease and smoking, immune suppression, obesity, and pregnancy, as well as chronic heart, kidney, and liver disease [7]. Increasing age was also found to be associated with higher risk of progression in those patients [8]. Nirmatrelvir 300 mg with ritonavir 100 mg is given as a five-day treatment course twice daily within five days of diagnosis [5]. Relapse of symptoms has been described recently; data are limited to some case reports at this time. A health advisory was issued in May of 2022 by CDC about the recurrence of symptoms in patients who are treated with Paxlovid. Symptoms are usually mild and resolve without additional treatment [4]. Our patients developed recurrence and worsening of their initial symptoms at diagnosis, and both patients improved with symptomatic management and without further antiviral treatment. The time between initial diagnosis and relapse in case reports was from two to eight days. In a case series of eight patients published in May of 2022, relapse symptoms in patients who have received ritonavir-boosted nirmatrelvir were reported 9-12 days following initial diagnosis [9]. The time from diagnosis to recurrence of symptoms was slightly longer in our patients: 11 and 14 days. Adverse reactions reported with the use of Paxlovid included dysgeusia, diarrhea, increase in serum creatinine, and increase in liver enzymes. Those adverse events were mild and resolved spontaneously [6]. Our patients reported no side effects following treatment with Paxlovid. Given the nature of the available data, it is unclear if the symptoms are due to progression of COVID-19 infection, relapse following treatment, or re-infection. More studies are required to further assess clinical characteristics, severity, and treatment, and to determine the cause of the emergence of the phenomenon.

## Conclusions

COVID-19 symptom rebound following treatment with ritonavir-boosted nirmatrelvir is a phenomenon that is being increasingly recognized recently. The data available are limited to case reports. The cases described had mild symptoms and required no further antiviral treatment. More studies are required to assess the clinical characteristics and severity in those patients and to determine the cause.
